# In vivo probing of SECIS-dependent selenocysteine translation in Archaea

**DOI:** 10.26508/lsa.202201676

**Published:** 2022-10-31

**Authors:** Nils Peiter, Michael Rother

**Affiliations:** Fakultät Biologie, Technische Universität Dresden, Dresden, Germany

## Abstract

Cotranslational insertion of selenocysteine (Sec) proceeds by recoding UGA to a sense codon. This recoding is governed by the Sec insertion sequence (SECIS) element, an RNA structure on the mRNA, but size, location, structure determinants, and mechanism differ for Bacteria, Eukarya, and Archaea. For Archaea, the structure–function relation of the SECIS is poorly understood, as only rather laborious experimental approaches are established. Furthermore, these methods do not allow for quantitative probing of Sec insertion. In order to overcome these limitations, we engineered bacterial β-lactamase into an archaeal selenoprotein, thereby establishing a reporter system, which correlates enzyme activity to Sec insertion. Using this system, in vivo Sec insertion depending on the availability of selenium and the presence of a SECIS element was assessed in *Methanococcus maripaludis*. Furthermore, a minimal SECIS element required for Sec insertion in *M. maripaludis* was defined and a conserved structural motif shown to be essential for function. Besides developing a convenient tool for selenium research, converting a bacterial enzyme into an archaeal selenoprotein provides proof of concept that novel selenoproteins can be engineered in Archaea.

## Abstract (precis)

By turning a bacterial reporter into an archaeal selenoprotein, in vivo probing of structure–function relations during UGA recoding for selenoprotein synthesis in Archaea is greatly facilitated.

## Introduction

The standard genetic code assigns 64 base triplets/codons to 20 canonical amino acids and three stop signals for protein biosynthesis termination (Nirenberg et al, 1966). The “21^st^” amino acid selenocysteine (Sec) is cotranslationally inserted into proteins by recoding UGA, which is normally a stop codon, that is, signaling termination of translation (Gesteland & Atkins, 1996). Sec-containing proteins (selenoproteins) are found in members of all three domains, Bacteria, Eukarya, and Archaea, but the trait of Sec synthesis and incorporation is not evenly distributed across the tree of life (Santesmasses et al, 2020). Although the general concept of tRNA-bound synthesis of Sec and its translational insertion via UGA recoding is conserved, significant differences exist for details of the respective pathways in the three domains. For example, only the archaeal and eukaryotic Sec synthetic pathways involve a phosphorylated aminoacyl intermediate (Carlson et al, 2004; Kaiser et al, 2005). For translation of Sec at dedicated UGA codons, a secondary structure on the selenoprotein mRNA, the Sec insertion sequence (SECIS) element (Berry et al, 1991), is common, although they are not similar to each other in sequence or structure across the domains. In Eukarya and Archaea, the SECIS is located in the 3′ untranslated region (3′-UTR) of the mRNA (Berry et al, 1991; Wilting et al, 1997; Rother et al, 2001); in Bacteria, it is directly 3′-adjacent to the UGA Sec codon (Zinoni et al, 1990). Also common to UGA recoding is a specialized translation elongation factor, SelB (designated as EFSec in eukaryotes), that binds the correctly aminoacylated tRNA specific for Sec (Sec-tRNA^Sec^) (Forchhammer et al, 1989; Fagegaltier et al, 2000; Rother et al, 2000; Tujebajeva et al, 2000). The ternary complex of SelB, GTP, and Sec-tRNA^Sec^ mediates communication between the recoding signal (the SECIS element) and the recoding site (UGA in the ribosomal A site) for Sec insertion. However, the mode of this communication differs between the domains. While bacterial SelB directly binds the SECIS (Baron et al, 1993), auxiliary factors, like the SECIS binding protein 2, SECp43, and ribosomal protein L30, form a recoding complex with EFSec (see Bulteau and Chavatte [2015] and references therein). For Archaea, details of the recoding mechanism are still unknown.

So far, the only members of the Archaea experimentally shown to contain Sec are methanogenic archaea (methanogens). Genomic analyses suggest that a recently proposed taxon, the Asgard archaea, also harbors members encoding Sec (Mariotti et al, 2016). Because this group is closely related to eukaryotes (Spang et al, 2019), the similarity of the Sec synthesis and incorporation machinery between the two domains may be a result of the vertical transfer from an archaeal ancestor (Mariotti et al, 2016). Thus, understanding the mechanism of Sec insertion in Archaea will help determining the relevance of this trait during the evolution of eukaryotes. Among the Archaea, *Methanococcus maripaludis* has become the prime model for studying selenoprotein synthesis. This is mainly due to its comparably fast growth, the comparably high sophistication—and number—of methods for genetic analysis (Sarmiento et al, 2011), and the non-essential nature of the Sec synthesis and incorporation machinery (Rother et al, 2003; Stock et al, 2011). Considering that most of the selenoproteins in methanogens are directly involved in their energy metabolism, methanogenesis (Rother & Quitzke, 2018), the latter was unexpected, but later explained by the presence of a Sec-independent alternative set of enzymes (Rother et al, 2003).

**Figure 1.**
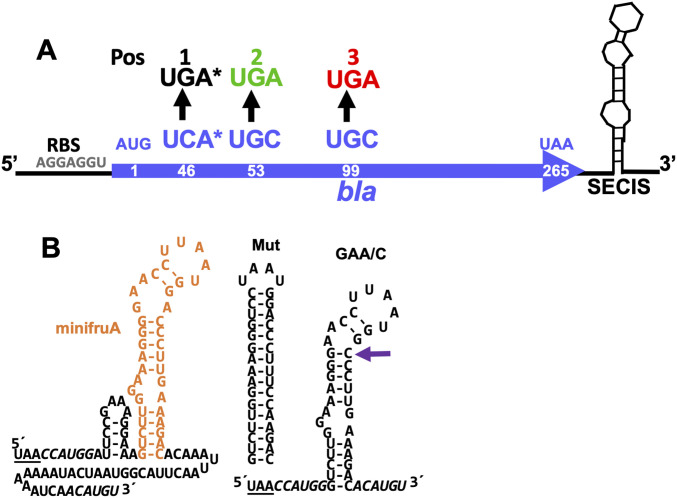
Construction of the β-lactamase (Bla) reporter. **(A)** General scheme of the reporter construct. RBS indicates the ribosome binding site in the 5′-UTR of the *sl* promoter; AUG, the start codon; and UAA, the stop codon. Black arrows indicate the codon exchange of UCA, UGC, and UGC, respectively, to UGA at three different positions (Pos 1, Pos 2, and Pos 3, respectively); asterisk indicates the codon for serine in the active site of Bla; the numbers within the coding region of *bla* indicate the amino acid position of the translated protein; the SECIS element variants are placed 3′ of the coding sequence of *bla*. **(B)** Predicted SECIS elements. Minimum free energy RNA structures were predicted using RNAfold (Lorenz et al, 2011); dashes indicate the Watson–Crick base pairing. 3′-UTR of *fruA* from *Methanococcus maripaludis* JJ is shown from TAA stop codon (underlined); the predicted minimal SECIS element of *fruA* (minifruA) (−9.5 kcal mol^−1^ minimal free energy, frequency 71.2%) is shown in orange; Mut is a completely based paired stem–loop with the same length as the minimal SECIS element (−30.3 kcal mol^−1^ minimal free energy, frequency 95.4%); GAA/C is a variant of the minimal SECIS element with one base exchange of A to C (indicated by purple arrow) (−12.3 kcal mol^−1^ minimal free energy, frequency 84.1%).

From analyzing putative Sec-encoding genes in methanogens (Wilting et al, 1997), a hypothetical consensus structure for the archaeal SECIS element was deduced. A basal helix of ca. 10 bp, sometimes harboring unpaired bases, with a G/C-rich apical end is followed by a highly conserved bulge, consisting of GAA opposed by A on the other side (GAA/A; Fig 1). Interestingly, this structure is reminiscent of the kink-turn motif found in SECIS elements of eukaryotes (Walczak et al, 1996; Klein et al, 2001). This archaeal kink-turn–like motif is followed by two or three G-C pairs, which lead into a non-conserved apical loop region of four to eight nucleotides (Rother & Quitzke, 2018). In only one previous study was the principal nature of the archaeal SECIS element experimentally addressed. There, it was shown that a secondary structure, previously predicted, was indeed part of a selenoprotein mRNA, that it attained the predicted structure in vivo, and that it was required for heterologous expression of a selenoprotein gene from *Methanocaldococcus jannaschii* in *M*. *maripaludis*, evidenced by the incorporation of radioactively labeled selenium (Rother et al, 2001).

The synthesis of selenoproteins can be assessed through the enzymatic activity of a natural selenoprotein, like formate dehydrogenase (Fdh) in *Escherichia coli* (Leinfelder et al, 1988) or deiodinase in eukaryotes (Berry et al, 1993), or through the direct detection of selenium (isotopes) in the protein (Heras et al, 2011). Development of an easily quantifiable reporter system, like the translational *fdhF*-*lacZ* fusion established in *E*. *coli* (Zinoni et al, 1987), was key for detailed structure–function analyses of the SECIS element and the UGA decoding event (Zinoni et al, 1990; Heider et al, 1992; Suppmann et al, 1999). In *M*. *maripaludis*, its formate-dependent growth behavior, its Fdh activity, and in vivo labeling of endogenous selenoproteins with [^75^Se] have been employed for analyzing Sec insertion (Rother et al, 2001). All these approaches are either laborious (due to the anaerobic nature of the organism), or “semi-quantitative,” or both. Developing a reporter system for methanogenic archaea where Sec insertion directly corresponds to (easily) quantifiable enzymatic activity would not only greatly facilitate assessing phenotypic consequences of mutant strains but also allow quantitatively probing structure–function relationships of factors involved in the process.

**Figure S1.**
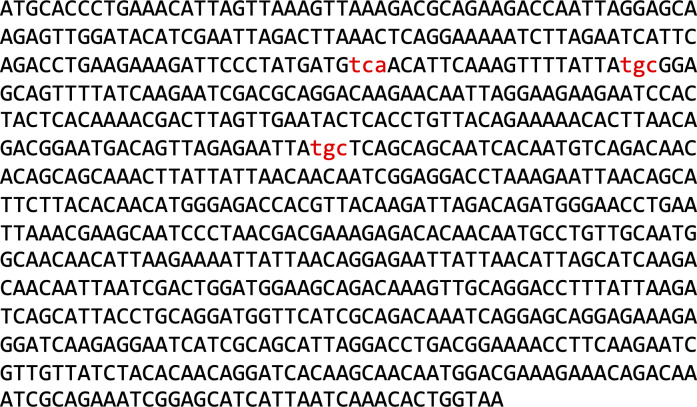
Primary sequence of the codon-optimized *bla* gene (without the signal peptide-encoding sequence). Lower-case letters (red) show the three codons exchanged for TGA.

In the present study, we engineered the class A (TEM) β-lactamase (Bla) from *E*. *coli* (Bla, accession number J01749.1) (264–amino acid residues, signal peptide omitted, 29.03 kD calculated mass; Fig S1) into a selenoprotein to study SECIS-dependent Sec translation in *M*. *maripaludis*. The enzyme is monomeric, contains no cofactor or posttranslational modification, is naturally active outside of the cell (i.e., robust), and is easily quantifiable with the chromogenic substrate nitrocefin. The enzyme hydrolyzes its substrate via a serine residue in the active site serving as a nucleophile to attack the β-lactam carbonyl (Tooke et al, 2019). By adding a SECIS encoding region to the *bla* gene, and by replacing three residues in Bla with Sec, fundamental conclusions about codon context requirements, about codon–SECIS distance limitations, and about selenium insertion efficiency could be drawn, thereby considerably extending our understanding of SECIS-dependent UGA recoding in Archaea.

## Results and Discussion

### Construction of a reporter for monitoring Sec insertion in Archaea

So far, only laborious and/or merely qualitative techniques for assessing Sec translation in Archaea are available. By establishing an easily quantifiable system, we sought to develop a method that allows probing of structure–function relations of the Sec translation apparatus, like the SECIS element. To this end, we engineered a translational reporter based on *bla* from *E*. *coli* (Fig 1A) shown to be actively produced in *M*. *maripaludis* (Quitzke et al, 2018). To achieve sufficient expression, the gene (lacking the signal peptide-encoding sequence) was placed under the control of a strong constitutive promoter (P*sl*) and a strong terminator (T*mcrA*). Three codons within the open reading frame of *bla* were individually changed to UGA in order to substitute the corresponding amino acids to Sec: the active site serine (S46U, Pos 1) and two cysteine residues (C53U, Pos 2; and C99U, Pos 3) (Fig 1A) shown to be involved in stability (through the formation of disulfide bonds) rather than required for enzymatic activity (Schultz et al, 1987). To direct Sec insertion during translation of these constructs, the 3′-UTR of *fruA* (MMJJ_14570) from *M*. *maripaludis* JJ, encoding the SECIS element of the gene for the Sec-containing large subunit of F_420_-dependent hydrogenase, was inserted (overlapping with a restriction site used for cloning) immediately downstream of the *bla* coding sequence (Fig 1B). Thus, either abundance or specific activity, or both, of Bla in the cells should depend on the functioning of the Sec insertion machinery, including UGA recoding by the *fruA* SECIS element.

### SECIS-dependent reporter activity

The three constructs, together with a wild-type (WT) variant of *bla* (i.e., without a Sec codon), were transferred to *M*. *maripaludis* JJ via a self-replicating shuttle vector (Lie & Leigh, 2003), which also constituted the vector control (VC) (i.e., lacking a *bla* reporter) (Table 1). In addition, reporter variants where the SECIS encoding sequence had been removed (-S) were also included (Table 1). Heterologous production of Bla in the respective strains was assessed via cleavage of nitrocefin in cleared cell lysates (see the Materials and Methods section).

**Table 1.** Plasmids used in this study.

Name	Relevant genotype/description/construction	Reference
pWLG40NZ-R	Amp^R^, Neo^R^, *Escherichia coli/Methanococcus maripaludis* shuttle vector	Lie and Leigh (2003)
pUC57-BsaI-free	Amp^R^, general cloning	Biocat.com
pUCblaWT	pUC57-BsaI-free, P*sl*-*bla-*3′-UTR_*fruA*_-T*_mcrA_* fusion	General Biosystems, Inc.
pUCblaWT-S	pUC57-BsaI-free, P*sl*-*bla-*T*_mcrA_* fusion, UTR-encoding sequence from pUCblaWT excised (NcoI, PciI) and religated	This study
pUCblaPos1	pUC57-BsaI-free, P*sl*-*bla-*3′-UTR_*fruA*_-T*_mcrA_* fusion with codon exchange at Position 1 (137C→G)	General Biosystems, Inc.
pUCblaPos1-S	pUC57-BsaI-free, P*sl*-*bla-*T*_mcrA_* fusion, UTR-encoding sequence from pUCblaPos1 excised (NcoI, PciI) and religated	This study
pUCblaPos2	pUC57-BsaI-free, P*sl*-*bla-*3′-UTR_*fruA*_-T*_mcrA_* fusion with codon exchange at Position 2 (159C→A)	General Biosystems, Inc.
pUCblaPos2-S	pUC57-BsaI-free, P*sl*-*bla-*T*_mcrA_* fusion, UTR-encoding sequence from pUCblaPos2 excised (NcoI, PciI) and religated	This study
pUCblaPos3	pUC57-BsaI-free, P*sl*-*bla-*3′-UTR_*fruA*_-T*_mcrA_* fusion with codon exchange at Position 3 (297C→A)	General Biosystems, Inc.
pUCblaPos3-S	Amp^R^, P*sl*-*bla-*T*_mcrA_* fusion, UTR-encoding sequence from pUCblaPos3 excised (NcoI, PciI) and religated	This study
pACYC177	Amp^R^, Kan^R^, general cloning	Chang and Cohen (1978)
pACYCblaPos3	Amp^R^, P*sl*-*bla-*3′-UTR_*fruA*_-T*_mcrA_* fusion from pUCblaPos3 subcloned via restriction/ligation to eliminate interfering restriction sites in the vector backbone	This study
pACYCblamut	Amp^R^, P*sl*-*bla-*3′-UTR_*fruA*_mut-T*_mcrA_* fusion, exchange of 3′-UTR from pACYCblaPos3 with 3′-UTR_*fruA*_mut	This study
pACYCblaminifruA	Amp^R^, P*sl*-*bla-*3′-UTR_*fruA*_mini-T*_mcrA_* fusion, exchange of 3′-UTR from pACYCblaPos3 with 3′-UTR_*fruA*_mini	This study
pACYCblaGAA/C	Amp^R^, P*sl*-*bla-*3′-UTR_*fruA*_miniGAA/C-T*_mcrA_* fusion, exchange of 3′-UTR from pACYCblaPos3 with 3′-UTR_*fruA*_miniGAA/C	This study
pEblaWT	Amp^R^, Neo^R^, P*sl*-*bla-*3′-UTR_*fruA*_-T*_mcrA_* fusion from pUCblaWT in pWLG40NZ-R	This study
pEblaWT-S	Amp^R^, Neo^R^, P*sl*-*bla-*T*_mcrA_* fusion from pUCblaWT without UTR sequence in pWLG40NZ-R	This study
pEblaPos1	Amp^R^, Neo^R^, P*sl*-*bla-*3′-UTR_*fruA*_-T*_mcrA_* fusion from pUCblaPos1 in pWLG40NZ-R	This study
pEblaPos1-S	Amp^R^, Neo^R^, P*sl*-*bla-*T*_mcrA_* fusion from pUCblaPos1 without UTR sequence in pWLG40NZ-R	This study
pEblaPos2	Amp^R^, Neo^R^, P*sl*-*bla-*3′-UTR_*fruA*_-T*_mcrA_* fusion from pUCblaPos2 in pWLG40NZ-R	This study
pEblaPos2-S	Amp^R^, Neo^R^, P*sl*-*bla-*T*_mcrA_* fusion from pUCblaPos2 without UTR sequence in pWLG40NZ-R	This study
pEblaPos3	Amp^R^, Neo^R^, P*sl*-*bla-*3′-UTR_*fruA*_-T*_mcrA_* fusion from pUCblaPos3 in pWLG40NZ-R	This study
pEblaPos3-S	Amp^R^, Neo^R^, P*sl*-*bla-*T*_mcrA_* fusion from pUCblaPos3 without UTR sequence in pWLG40NZ-R	This study
pEblamut	Amp^R^, Neo^R^, P*sl*-*bla-*3′-UTR_*fruA*_mut-T*_mcrA_* fusion from pACYCblamut in pWLG40NZ-R	This study
pEblaminifruA	Amp^R^, Neo^R^, P*sl*-*bla-*3′-UTR_*fruA*_mini-T*_mcrA_* fusion from pACYCblaminifruA in pWLG40NZ-R	This study
pEblaGAA/C	Amp^R^, Neo^R^, P*sl*-*bla-*3′-UTR_*fruA*_miniGAA/C-T*_mcrA_* fusion from pACYCblaGAA/C in pWLG40NZ	This study

**Figure 2.**
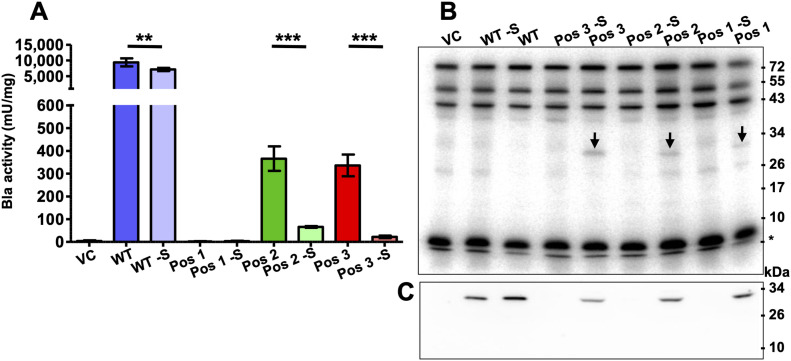
SECIS-dependent Sec insertion into Bla. **(A)** Specific activity of Bla in cleared cell lysates of *M. maripaludis* carrying variants Pos 1, Pos 2, and Pos 3, with and without (-S) the 3′-UTR of *fruA* of *M. maripaludis* containing the predicted SECIS element; WT, *bla* without a UGA codon; the empty vector pWLG40NZ-R without the reporter construct (VC) was used as control; error bars show a 95% confidence interval of at least four biological replicates; comparison was performed by an unpaired two-tailed *t* test. **P < 0.01 and ***P < 0.001; experiments were reproduced at least once. **(B)** Sec insertion into Bla. Autoradiograph of a 10% PAGE gel after electrophoresis of ^75^Se-labeled lysates (see the Materials and Methods section) of *M. maripaludis* cells carrying the reporter constructs (see panel A); experiment was reproduced at least once. **(C)** Synthesis of Bla. Cleared lysates of cells carrying the reporter constructs (see panel A) containing 10 µg protein, except for WT and WT-S where 1/10 was applied, were immunoblotted with α-Bla antisera (see the Materials and Methods section); experiment was reproduced at least once.

The Bla activity of the VC was considered the background noise (4.2 ± 4.5 mU mg^−1^; Table S1). That of the WT variant, irrespective of whether the SECIS element was present or not, was substantial (between more than 7,000 and 9,000 mU; Fig 2A and Table S1). When the codon for the active site serine was replaced for a Sec codon (Pos 1), Bla activity corresponded to that of the VC, again, irrespective of whether the SECIS element was present or not (Fig 2A). In contrast, Bla activity of variants Pos 2 and Pos 3 clearly depended on whether the 3′-UTR (containing the predicted SECIS element) was present on the mRNA. When absent, activity ranged between ∼65 (Pos 2) and 20 (Pos 3) mU mg^−1^ (Table S1), which is well discernible from the background noise (Fig 2A and Table S1). When the SECIS element was present, Bla activity was more than fivefold and 15-fold higher, respectively, in the range of 350 mU mg^−1^ (Fig 2A and Table S1). Thus, suppression of an UGA codon reduces Bla activity at least 20-fold compared with the WT allele (Table S1). The SECIS-independent Bla activity observed for Pos 2 and Pos 3 was less than 1% of that for WT, which is in the same range that resulted from Sec-independent UGA suppression during the synthesis of Fdh in *M*. *maripaludis* (Seyhan et al, 2015). However, most of the translated UGA-containing *bla* mRNA depends on the presence of a SECIS element, which strongly indicates that Sec is inserted at the respective positions.

Table S1 Reporter activity measurements in *Methanococcus maripaludis* JJ.

### Engineering an archaeal selenoprotein

**Figure S2.**
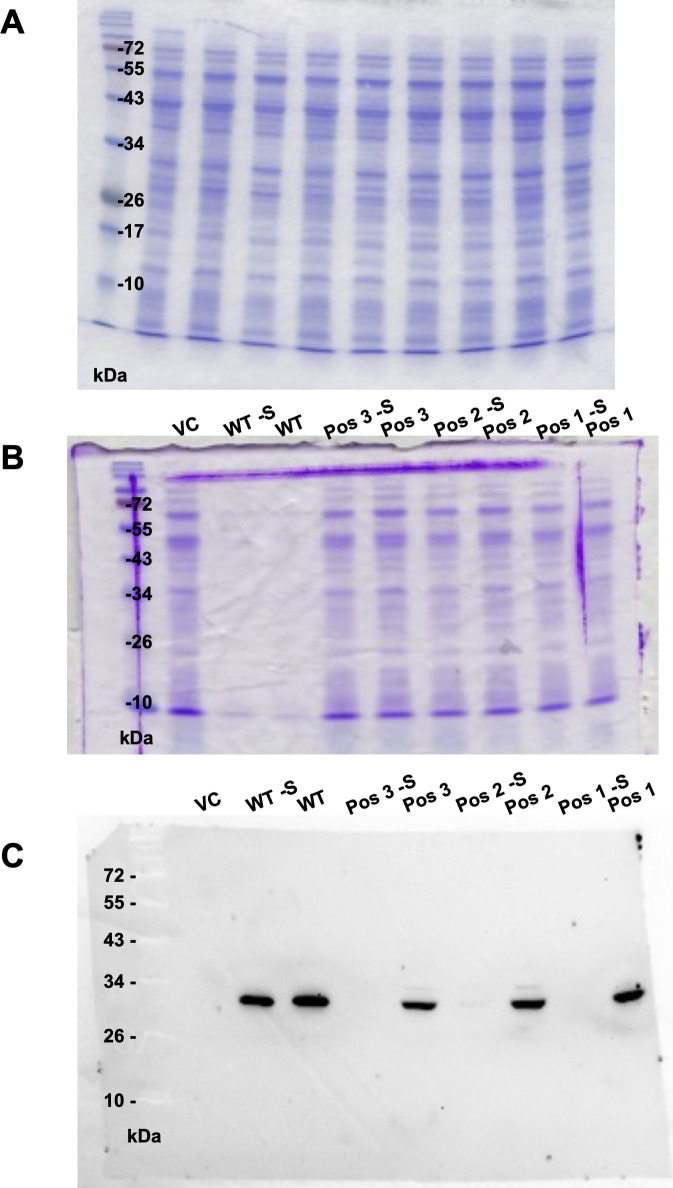
PAGE gels and blots I. **(A)** Coomassie-stained PAGE gel containing ^75^Se-labeled macromolecules used for autoradiography as seen in Fig 2B; **(B)** Coomassie-stained PAGE gel prepared in parallel to the one used for blotting as seen in Fig 2C; **(C)** immunoblot shown in Fig 2C, only overexposed for 45 min.

To unambiguously demonstrate cotranslational Sec insertion into Bla via the archaeal selenoprotein synthesis machinery, the strains carrying the reporter constructs were metabolically labeled with radioactive selenium (^75^Se-selenite; see the Materials and Methods section). Beside the known selenoproteins (Fig 2B, VC), *M*. *maripaludis* synthesized another Sec-containing macromolecule electrophoretically migrating at ∼30 kD, but only when *bla* contained UGA, and only when the mRNA contained the 3′-UTR SECIS region (Fig 2B, arrow). Notably, the Pos 1 variant also contained Sec, despite the fact that it was not active. In members of this class of Bla, the active site serine acts as the reaction nucleophile and hydrolyzes β-lactams via a covalent acyl-enzyme intermediate (Tooke et al, 2019). Replacing the active site serine with another nucleophile, cysteine, resulted in active enzyme but >10-fold reduced affinity toward nitrocefin (Sigal et al, 1984). Despite the fact—or maybe because—nucleophilicity of Sec is even higher than that of cysteine (Arner, 2010), Sec can apparently not functionally replace serine in this context. The electrophoretic behavior strongly suggested that the new selenoprotein of *M*. *maripaludis* is Bla. To confirm this notion, commercially available polyclonal antibodies against Bla from *E*. *coli* were used to probe cell extracts of the strains (see the Materials and Methods section). Only one specific signal was observed (Fig 2C) (except when markedly overexposed; Fig S2), again electrophoretically migrating at ∼30 kD, which corresponds to the predicted mass of Bla (29.03 kD). The lack of detection for the Pos 2 and Pos 3 variants without the SECIS, despite the fact that they showed some residual activity, is probably due to their abundance being close to, or below, the detection limit of the antiserum (Fig S2). The fact that no Bla fragments with lower mass were detected indicates that the protein truncated at Pos 3 (∼11.1 kD; the other variants would not be resolved by the gel system used here) is rapidly degraded. The WT variant (not containing Sec) was much more abundant in cell extract than any of the Sec-containing variants, which is consistent with the Bla activities in the corresponding strains (compare Fig 2A and C). Whether the reduced abundance of the Pos 2 and Pos 3 variants, compared with the WT, is due to exchanging a cysteine residue involved in Bla stability, or due to the inherently slow and inefficient insertion of Sec (Suppmann et al, 1999), remains to be demonstrated.

### Expression of Sec-encoding *bla*

**Figure 3.**
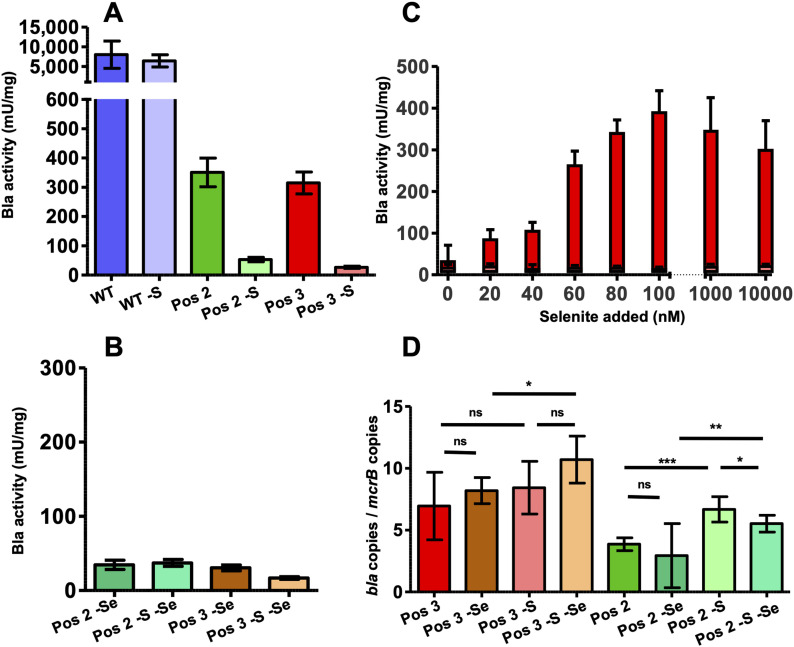
Characteristics of the Sec-containing Bla reporter. **(A)** Stability of Bla. Specific activity was determined like in Fig 2A, but only after 10 consecutive transfers of the respective cultures. **(B)** Selenium dependence of Bla activity. Specific activity was determined in cleared lysates of cells (see Fig 2A) grown (at steady state) in the absence of added selenium; an unpaired two-tailed *t* test was performed comparing the respective activity in the same constructs with selenite present (Fig 2A); error bars show a 95% confidence interval of at least four biological replicates; experiment was reproduced at least once. **(C)** Titration of Bla activity with selenite. Specific activity was determined in cleared lysates of variants Pos 3 (red) and Pos 3-S (light red), grown (at steady state) in a medium where selenite, to the concentrations indicated, had been added; error bars show a 95% confidence interval of at least three biological replicates; experiment was reproduced at least once. **(D)** Abundance of *bla* mRNA. Samples correspond to cultures in Fig 2, panel A; and Fig 3, panel B; error bars show a 95% confidence interval of at least three biological replicates; experiment was reproduced at least once; comparison was performed by an unpaired two-tailed *t* test. ns, not significant. *P < 0.05; **P < 0.01; and ***P < 0.001.

Expression of the engineered selenoprotein gene is governed by a strong constitutive promoter. Such strong and constant expression signal bears the risk of degeneration, that is, to be lost over time through mutation in order to eliminate a genetic/metabolic load not conferring selective advantage (Glick, 1995). To demonstrate a reliable correlation between the SECIS-dependent Sec insertion during *bla* translation and the activity of the resulting enzyme, it was quantified as before (see Fig 2A, except for the VC and the Pos 1 variant), but only after the cultures had been transferred 10 times (2% from late exponential growth phase into fresh medium, corresponding to ∼50 generations) without relief of antibiotic selection. In none of the strains did Bla activity markedly decrease, which confirmed the stability and durability of the reporter system (compare Figs 2A and 3A and Table S1).

As Bla activity could be linked to the SECIS-dependent Sec insertion in variants Pos 2 and Pos 3, the amount of selenium available to the Sec synthesis and insertion machinery of *M*. *maripaludis* should affect the readout of the reporter. To test this notion, both variants Pos 2 and Pos 3, each with and without the SECIS, were cultivated for three passages on medium to which no selenium had been added. Although nothing is known as to how *M*. *maripaludis* transports selenium into the cell, or if the element can be intracellularly accumulated and/or stored, this measure is appropriate to establish steady-state conditions (Quitzke et al, 2018). In the absence of added selenium, the SECIS-dependent Bla activity dropped ∼10-fold to the respective levels of the reporter variants lacking the SECIS element (Fig 3B), again strongly suggesting that the remaining activity stems from Sec-independent UGA suppression. The drop in Bla activity when selenium was omitted from the growth medium carrying the reporter prompted us to investigate the selenium dependence further. To this end, variant Pos 3 was grown (to a steady state) in the presence of various concentrations of selenite. The resulting Bla activity correlated with the selenium status of the cells between 20 and 100 nM, and this correlation depended again on the presence of the SECIS element (Fig 3C). Beyond 100 nM selenite, Bla activity did not increase further (Fig 3C).

We noted a minor but significant difference in Bla activity in the two WT constructs, which indicated that the SECIS element might stabilize the mRNA somewhat (Fig 2A). To confirm that the SECIS element exerts no effect other than directing Sec insertion (its feature under study here), the mRNA abundance of *bla* mRNA was quantified in strains carrying variants Pos 2 and Pos 3, with and without the SECIS element, respectively, and grown in the presence or absence of added selenium. The presence of a SECIS element did not increase the amount of the respective mRNA, that is, did not increase its half-life, regardless of whether selenium was present in the growth medium of the respective strain or not (Fig 3D and Table S2). Thus, the Bla activities observed in this study are not affected by differences in *bla* mRNA abundance. It might be worth noting that bacterial mRNAs containing “premature” stop codons are degraded rapidly (Morse & Yanofsky, 1969), which was not observed here. The basis for this phenomenon, and for the ca. twofold difference in mRNA abundance between variants Pos 2 and Pos 3, is not known.

Table S2 mRNA abundance in *Methanococcus maripaludis* JJ constructs.

Taken together, the data presented establish that *bla* mRNA containing UGA is translated into a selenoprotein and that Sec insertion depends on the presence of a SECIS element in the 3′-UTR and on the presence of selenium for the synthesis of Sec. Beyond providing a quantifiable and facile tool, this system allows fundamental insights into the physiology of *M*. *maripaludis* and the mechanism of selenoprotein synthesis in Archaea: Sec insertion into the engineered selenoprotein appears to be saturated between 0.1 and 1 *µ*M selenite in the medium (Fig 3C), which is in the same range as that reported for selenium-dependent transcriptional regulation in *M*. *maripaludis* (Quitzke et al, 2018). Furthermore, the synthesis of Sec-containing Bla did not affect the abundance of the other selenoproteins of *M*. *maripaludis* visible through metabolic labeling (Fig 2B), which suggests that the Sec synthesis and incorporation machinery of the organism has sufficient capacity.

### Structure–function relation of archaeal SECIS elements

**Figure 4.**
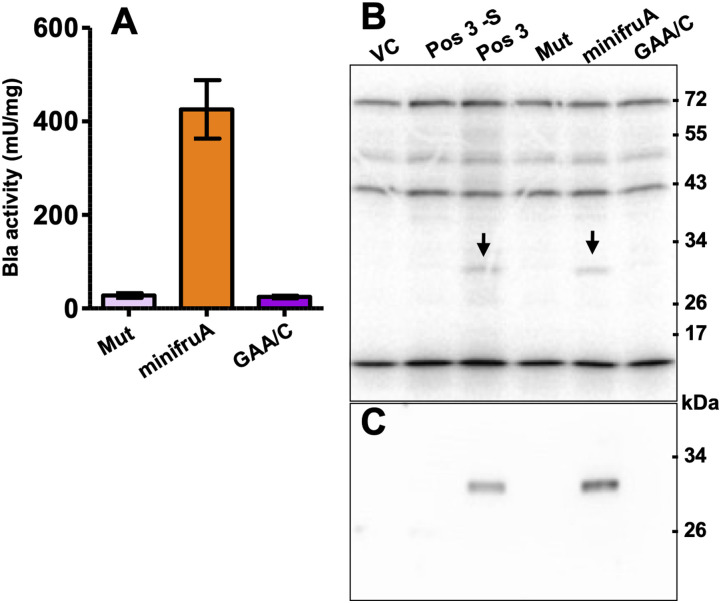
Probing structure-function relations of the archaeal SECIS element. **(A)** Specific activity of Bla in cleared cell lysates of *M. maripaludis* carrying variants Mut, minifruA, and GAA/C; variant Pos 3 and Pos 3-S were used as controls; error bars show a 95% confidence interval of at least four biological replicates; experiment was reproduced at least once. **(B)** Sec insertion into Bla. Autoradiograph of a 10% PAGE gel after electrophoresis of ^75^Se-labeled lysates (see the Materials and Methods section) of *M. maripaludis* cells carrying the reporter constructs (see panel A); the empty vector pWLG40NZ-R without the reporter construct was used as control (VC); experiment was reproduced at least once. **(C)** Synthesis of Bla. Cleared lysates of cells carrying the reporter constructs (see panel B) containing 10 µg protein were immunoblotted with α-Bla antisera (see the Materials and Methods section); experiment was reproduced at least once.

**Figure S3.**
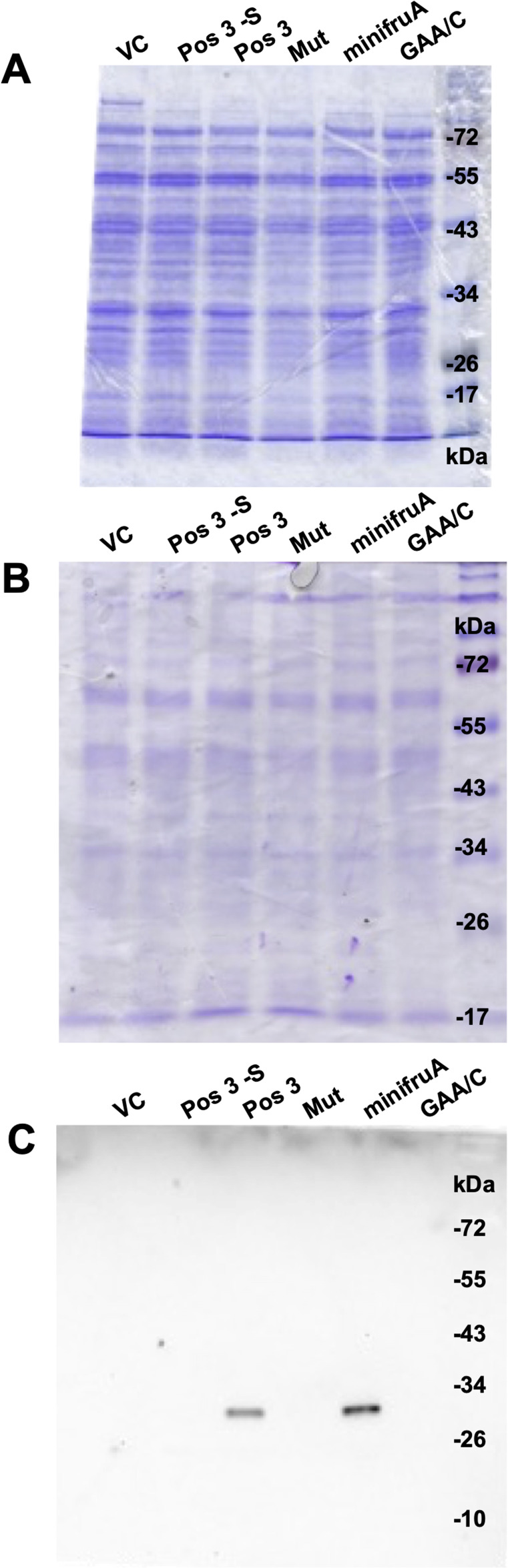
PAGE gels and blots II. **(A)** Coomassie-stained PAGE gel containing ^75^Se-labeled macromolecules used for autoradiography as seen in Fig 2B; **(B)** Coomassie-stained PAGE gel prepared in parallel to the one used for blotting as seen in Fig 4C; **(C)** immunoblot shown in Fig 4C, only overexposed for 45 min.

**Figure S4.**
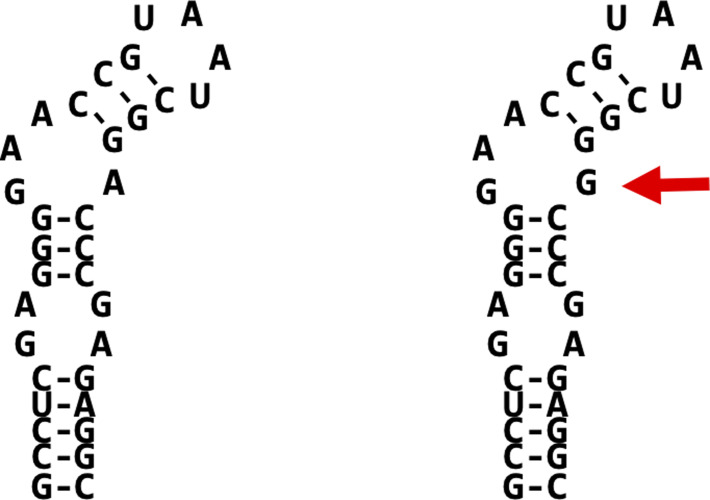
SECIS elements from *Methanocaldococcus jannaschii* analyzed previously. Comparison between the predicted WT *fruA* SECIS element (left) and the fruAGAA/G SECIS variant leading to a 75% reduction in function (Rother et al, 2001). Two similar minimum free energy RNA structures were predicted using RNAfold (Lorenz et al, 2011); dashes indicate the Watson–Crick base pairing (WT, −19.5 kcal mol^−1^ minimal free energy, frequency 48.3%; fruAGAA/G, −19.5 kcal mol^−1^ minimal free energy, frequency 86.9%; compare this structure with the GAA/C variant analyzed in Fig 1B).

After characterizing the synthesis of Sec-containing Bla, thereby confirming its usefulness as a proxy for directly monitoring Sec insertion in methanogenic archaea, the mechanism of SECIS-dependent UGA recoding was investigated further. The Pos 3 variant of *bla* was chosen as the reporter, as it showed the largest difference between “no activity” (i.e., without the SECIS element, without selenium) and “full activity” (i.e., with the SECIS element, at 1 *µ*M selenite). To assess the SECIS variant functionality, Bla activity, Bla synthesis, and ^75^Se incorporation were assessed (Figs 4 and S1). First, a SECIS variant constituting a symmetric fully base-paired stem–loop lacking the GAA/A-motif (Mut; Fig 1B), was analyzed. No Bla activity (Fig 4A), no Sec incorporation (Fig 4B), and no Bla synthesis (Figs 4C and S3) could be observed in the presence of a Mut-SECIS, which shows that a mere stem–loop is not sufficient, and that the motif removed is critical, to act as a SECIS element. The same principal result was obtained when Sec insertion into FruA of *M*. *jannaschii* was studied by metabolic labeling (Rother et al, 2001). Second, the *fruA* SECIS region was shortened to more rigorously define it. The 5′-region was shortened by 15 nucleotides (thereby eliminating a predicted small 5′-stem–loop; Fig 1B), and from the 3′-region, 33 nucleotides were removed, generating a “minimal” SECIS element, “minifruA” (Fig 1B). Sec insertion into Bla mediated by minifruA was no less than with the original SECIS encoding region. Thus, the archaeal SECIS element could be experimentally confined, that is, defined by means other than sequence identity/similarity. Third, a SECIS variant was used, where in the GAA/A-motif, a presumed critical region, a single nucleotide was exchanged (“GAA/C”). Indeed, this measure sufficed to eliminate the function of the element as SECIS completely (Fig 4). Interestingly, when the same position of the *M*. *jannaschii fruA*–SECIS was changed to guanine (A→G), a seemingly milder impairment of Sec insertion (∼75% reduction), assessed by metabolic labeling, was observed (Rother et al, 2001). A possible explanation is that when cytosine (A→C) is present at this position, the 5′-G of the GAA/A motif would base pair to it, essentially dissolving the kink-turn–like structure (Fig 1B), while retaining it when G is present opposite the GAA (Fig S4). Possibly, the flanking (G-C) base pairs are meant to stabilize the GAA/A motif, which is why exchanging the distal (of three) G-C to A-U had only a mild effect (Rother et al, 2001). Thus, unlike the situation for bacterial and eukaryal Sec insertion (Heider et al, 1992; Martin et al, 1998), structural rather than sequence identities are functional determinants of archaeal SECIS elements.

Here, we showed that the SECIS element in the 3′-UTR facilitates (with similar apparent efficiencies) Sec insertion at three different positions in Bla (Fig 2B), arguing against strict limitations of the distance between the recoding signal (the SECIS element) and the site of recoding (the UGA translating ribosome). In *fruA* of *M*. *maripaludis*, the distance between the UGA and the SECIS element (5′-base of the stem) is 88 nucleotides (Quitzke et al, 2018); in Pos 1, 2, and 3 of Sec-containing Bla, it is 679, 658, and 520 nucleotides, respectively, which is well within the range deduced for archaeal selenoprotein genes (Quitzke et al, 2018). Furthermore, the SECIS element can be moved in the 5′-direction to directly follow the coding region of the selenoprotein gene without any apparent impairment of its function. Thus, Sec insertion resembles the system in eukaryotes not only in terms of the SECIS location but also in terms of lacking stringent constraints for the distance to the Sec codon (Berry et al, 1993). That Bla activity is proportional to the amount of available selenium highlights the use of this reporter for quantitative probing. Once the factor(s) binding the SECIS during UGA recoding is (are) identified, its (their) interaction(s) can be studied in vivo in detail.

**Figure S5.**
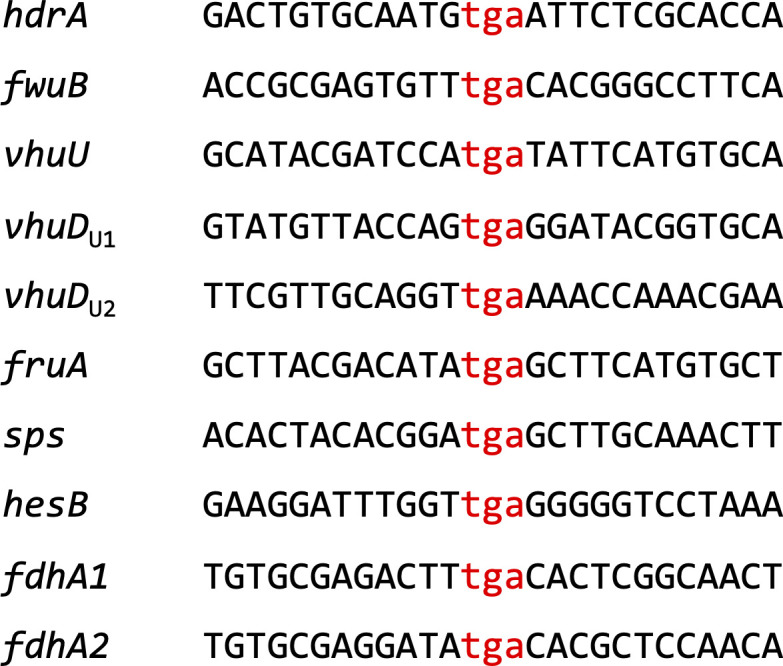
Vicinity of Sec (TGA) codons in the nine selenoprotein genes of *Methanococcus maripaludis* JJ (note that *vhuD* contains two TGA codons); for details, see Rother and Quitzke (2018).

Lastly, three endogenous amino acids could be exchanged with Sec arguing against stringent requirements for codon context in Archaea. Even the natural Sec codons in *M*. *maripaludis* have no apparent base context in their vicinity (Fig S5). In this feature, archaeal Sec insertion again resembles the eukaryal system more than the bacterial system, where the SECIS element itself represents a dramatic context constraint (Heider et al, 1992). Considering the degree of apparent flexibility, the selenoprotein gene expression system reported here will therefore not only aid in unraveling the mechanism of SECIS-dependent Sec insertion in methanogenic archaea, but may also allow to engineer novel selenoproteins with novel properties (Boschi-Müller et al, 1998), particularly ones requiring reducing and/or anaerobic conditions.

## Materials and Methods

### Strains and growth conditions

Strains of *Escherichia* *coli* were grown under standard conditions and transformed with plasmid DNA by electroporation (Sambrook et al, 1989). Where appropriate, 100 *µ*g ml^−1^ ampicillin was added to the medium for the selection of plasmids conferring the corresponding resistance. *M*. *maripaludis* strain JJ (DSMZ 2067) (Jones et al, 1983) was cultivated in McSe medium containing 10 mM sodium acetate (Rother et al, 2003). When selecting for pWLG40NZ-R (Lie & Leigh, 2003) and derivatives of it, 0.5 mg ml^−1^ (agar plates) or 1 mg ml^−1^ (liquid culture) neomycin was present in the medium, including the experiments addressing reporter stability. To generate selenium-adequate conditions, sodium selenite was added from a sterile anaerobic stock solution to a final concentration of 1 *µ*M. For lower concentrations, the medium containing selenite was diluted with McSe medium. Cultures were pressurized with 2 × 10^5^ Pa of H_2_:CO_2_ (80:20), which served as the sole energy source, and incubated at 37°C with gentle agitation. Growth was monitored photometrically at 578 nm (OD_578_) using a Genesys 20 spectrophotometer (Thermo Fisher Scientific).

Transformation and plating of *M*. *maripaludis* was conducted as described previously (Stock et al, 2010). In vivo labeling of *M*. *maripaludis* with [^75^Se]-selenite and analysis of the selenoproteome were basically conducted as described (Stock et al, 2010). Briefly, *M*. *maripaludis* was grown in the medium supplemented with Na-[^75^Se]-selenite (Eckert & Ziegler) to a final activity of 37 kBq ml^−1^ (specific activity of 37 GBq mmol^−1^). After harvesting and washing with McSe medium by centrifugation, cells were lysed in water containing 1 *µ*g ml^−1^ DNase I and 1 *µ*g ml^−1^ RNase A. Cell debris was sedimented by centrifugation. Proteins in the supernatant (cleared lysate) were separated by discontinuous denaturing PAGE (SDS–PAGE) (Laemmli, 1970). Autoradiography was conducted by Phosphoimaging using a phosphor screen and the Typhoon Trio (GE Healthcare). Migration positions of labeled macromolecules were compared with those of reference proteins (Color Prestained Protein Standard, Broad Range; New England Biolabs).

### Molecular methods and cloning

Standard molecular methods were used for the manipulation of plasmid DNA from *E*. *coli* DH10B (Sambrook et al, 1989). Plasmids used in this study are listed in Table 1. All DNA fragments derived from PCR (oligonucleotides used are listed in Table S3) and used for cloning were sequenced by Microsynth Seqlab using the BigDye Terminator Cycle Sequencing protocol. Reporter cassettes in pACYC177 derivatives were amplified with PCR to increase the amount of DNA for cloning into pWLG40NZ-R. The principal reporter construct, flanked by XhoI and BglII restriction sites, respectively, consists of the 5′-region of the S-layer–encoding structural gene (*sla*) of *Methanococcus voltae* (Kansy et al, 1994) overlapping the start codon of the *bla* gene, codon-optimized for *M*. *maripaludis* (Quitzke et al, 2018), the sequence encoding the 3′-UTR of *M*. *maripaludis* JJ *fruA* (MMJJ_14570), and the transcription terminator of *mcrA* of *M*. *voltae* (Müller et al, 1985) (Fig 1A). Four variants of the principal reporter were synthesized (General Biosystems, Inc.): the WT and three variants where a different codon within *bla* was exchanged for TGA (Sec/stop) codons resulting in the constructs Pos 1 (S46U), Pos 2 (C53U), and Pos 3 (C99U). The fragment for the 3′-UTR of *fruA* was exchanged after moving the reporter construct to pACYC177 via restriction cloning using HindIII and XhoI, which was done to eliminate interfering restriction sites in the vector backbone. To exchange regions for 3′-UTRs through restriction cloning (NcoI/PciI), double-stranded oligonucleotides were used. To this end, two complementary oligonucleotides (Table S3) containing appropriate overhangs suitable for restriction cloning were annealed, subsequently 5′-phosphorylated using T4 polynucleotide kinase (Thermo Fisher Scientific), and purified with the aid of illustra MicroSpin G-25 columns (GE Healthcare) before ligation. Reporter constructs were moved to pWLG40NZ-R via restriction cloning using XhoI and BglII, except for pEblaWT and pEblaGAA/C (Table 1), which were amplified via PCR (Table S3) before Gibson cloning (Gibson, 2011). The resulting episomal reporter plasmids were used to transform *M. maripaludis* JJ.

Table S3 Oligonucleotides used in this study.

### Quantification of Bla

Bla activity in *M. maripaludis* JJ carrying the reporter plasmids was quantified with nitrocefin (Biomol) as described (Quitzke et al, 2018), except that cells were harvested at an OD_578_ of ∼0.3. Bla activity, determined at 486 nm using a molar extinction coefficient of 20,500 M^−1^ cm^−1^, is expressed as milliunits (mU) per mg protein (1 U = 1 μmol nitrocefin cleaved per min). To convert mU into the SI unit nkat, values are multiplied by 0.016. Protein in cell fractions was quantified with the method of Bradford (Bradford, 1976) using bovine serum albumin as standard.

### Quantification of mRNA

Quantification of *M. maripaludis* mRNA was conducted via reverse transcription quantitative PCR (RT-qPCR). Cells were harvested by centrifugation from 2 ml of culture (OD_578_ ca. 0.3). If not used directly, cell pellets were snap-frozen in liquid nitrogen and stored at −80°C until use. RNA was isolated from the cells using the High Pure RNA Tissue Kit (Roche), lysing cells in lysis/binding buffer for 10 min on ice, and following the manufacturer’s instructions including on-column DNase treatment. Eluted RNA preparations were either directly used for the synthesis of cDNA, with gene-specific oligonucleotides (Table S3), or stored at −80°C for later use. The absence of DNA was confirmed via qPCR. The synthesis of cDNA and qPCR, and analysis of the data were conducted as described (Stock et al, 2011), except that for cDNA synthesis, the SuperScript III Reverse Transcriptase (Invitrogen) was used and that for qPCR, the Luna Universal qPCR Master Mix (New England Biolabs) with the qTOWER³ (Analytik Jena) was used. Also, treatment with RNase H after cDNA synthesis was omitted. Specific oligonucleotides (Table S3) for *bla* were designed with the help of Primer3Plus (Untergasser et al, 2007). For cDNA synthesis of *mcrB*, encoding the β-subunit of methyl-coenzyme M reductase, the oligonucleotide used is specific for the allele from strain S2 (MMP1555) (Stock et al, 2011) and has one base difference to the corresponding sequence of strain JJ (MMJJ_12810; Table S3). The data were analyzed and normalized to the expression of the *mcrB* as described (Stock et al, 2011). Amounts of mRNA *bla* copy numbers are shown per mRNA copy number of *mcrB*.

### Immunoblot analysis

For electrophoretic separation of *M. maripaludis* proteins, 5 ml of culture was harvested by centrifugation and the cells were resuspended in lysis solution (1 *µ*g ml^−1^ DNase I and 1 *µ*g ml^−1^ RNase A in water). Separation of cell debris by centrifugation was conducted at 14,000*g* for 10 min. Separation of proteins in the cleared supernatant via SDS–PAGE and their immunodetection were carried out as described (Oelgeschläger & Rother, 2009), except that the transfer of proteins onto nitrocellulose membranes was achieved by tank blotting in transfer buffer (190 mM glycine, 25 mM Tris base, and 20% [vol/vol] methanol, pH 8.3) (Schmid & Böck, 1984), using a Mini Trans-Blot cell (Bio-Rad) for 1 h at 120 V. For immunodetection, a commercial anti-β-lactamase (α-Bla) polyclonal antibody (AB3738-I; Merck KGaA) was used at a 1:1,000 dilution with a protein A–horseradish peroxidase conjugate (Bio-Rad) as the secondary antibody. Detection was carried out as described (Oelgeschläger & Rother, 2009), except that instead of an X-ray film, the Fusion FX imager (Vilber Lourmat) was used. Migration signals were compared with those of reference proteins (Color Prestained Protein Standard, Broad Range; New England Biolabs).

### Statistical analysis

The comparison between two cohorts with an unpaired two-tailed *t* test and other statistical analyses were conducted using GraphPad Prism version 5.03 (GraphPad Software).

## Supplementary Material

Reviewer comments
