# Supplementary material for: In vivo probing of SECIS-dependent selenocysteine translation in Archaea
Source: Life Sci Alliance. 2022 Oct 31;6(1):e202201676. doi: 10.26508/lsa.202201676 (PMC9622424; doi:10.26508/lsa.202201676)
Supplement: Supplementary file 1 [file LSA-2022-01676_TableS1.docx]

Supplementary Table S1: Reporter activity measurements in *M. maripaludis* JJ

| Plasmid^a^ | Construct | Selenium status^b^ | #Replicates | Bla activity^c^ | ± 95% confidence interval |
| --- | --- | --- | --- | --- | --- |
| pWLG40NZ-R | VC | 1 | 4 | 4.2 | 4.5 |
| pEblaWT | WT | 1 | 5 | 9,388.6 | 1,258.1 |
| pEblaWT^d^ | WT | 1 | 4 | 8,009.6 | 3,482.0 |
| pEblaWT-S | WT -S | 1 | 5 | 7,122.2 | 504.7 |
| pEblaWT-S^d^ | WT -S | 1 | 4 | 6,426.5 | 1,550.1 |
| pEblaPos1 | Pos 1 | 1 | 4 | 2.4 | 1.1 |
| pEblaPos1-S | Pos 1 -S | 1 | 4 | 3.6 | 2.7 |
| pEblaPos2 | Pos 2 | 1 | 4 | 365.9 | 53.8 |
| pEblaPos2^d^ | Pos 2 | 1 | 6 | 350.8 | 49.1 |
| pEblaPos2-S | Pos 2 -S | 1 | 4 | 66.1 | 3.6 |
| pEblaPos2-S^d^ | Pos 2 -S | 1 | 6 | 52.9 | 7.5 |
| pEblaPos3 | Pos 3 | 1 | 6 | 336.1 | 47.6 |
| pEblaPos3^d^ | Pos 3 | 1 | 5 | 314.8 | 37.6 |
| pEblaPos3-S | Pos 3 -S | 1 | 6 | 22.1 | 5.3 |
| pEblaPos3-S^d^ | Pos 3 -S | 1 | 6 | 26.5 | 3.8 |
| pEblaPos2 | Pos 2 | 0 | 4 | 34.7 | 6.2 |
| pEblaPos2-S | Pos 2 -S | 0 | 4 | 37.2 | 4.6 |
| pEblaPos3 | Pos 3 | 0 | 5 | 30.5 | 3.9 |
| pEblaPos3-S | Pos 3 -S | 0 | 6 | 16.8 | 1.7 |
| pEblaPos3 | Pos 3 | 0 | 3 | 30.0 | 36.3 |
| pEblaPos3 | Pos 3 | 0.02 | 7 | 82.6 | 20.9 |
| pEblaPos3 | Pos 3 | 0.04 | 4 | 103.0 | 18.0 |
| pEblaPos3 | Pos 3 | 0.06 | 5 | 260.5 | 31.3 |
| pEblaPos3 | Pos 3 | 0.08 | 5 | 338.1 | 28.5 |
| pEblaPos3 | Pos 3 | 0.1 | 6 | 387.7 | 60.8 |
| pEblaPos3 | Pos 3 | 1 | 4 | 343.1 | 77.1 |
| pEblaPos3 | Pos 3 | 10 | 4 | 297.2 | 36.3 |
| pEblaPos3-S | Pos 3 -S | 0 | 3 | 19.9 | 13.1 |
| pEblaPos3-S | Pos 3 -S | 0.02 | 5 | 22.6 | 4.9 |
| pEblaPos3-S | Pos 3 -S | 0.04 | 4 | 16.2 | 13.0 |
| pEblaPos3-S | Pos 3 -S | 0.06 | 4 | 19.4 | 4.7 |
| pEblaPos3-S | Pos 3 -S | 0.08 | 4 | 19.3 | 2.6 |
| pEblaPos3-S | Pos 3 -S | 0.1 | 6 | 16.9 | 1.4 |
| pEblaPos3-S | Pos 3 -S | 1 | 4 | 22.1 | 5.0 |
| pEblaPos3-S | Pos 3 -S | 10 | 4 | 23.6 | 13.1 |
| pEblaPos3mut | Mut | 1 | 6 | 27.8 | 4.9 |
| pEblaPos3minifruA | minfruA | 1 | 4 | 425.8 | 62.5 |
| pEblaPos3minifruA_A_C | GAA/C | 1 | 4 | 24.6 | 2.9 |

a: see Table 1

b: selenite added to the medium (µM)

c: mU mg^-1^,
mean

d: after 10 transfers
