# Supplementary material for: In vivo probing of SECIS-dependent selenocysteine translation in Archaea
Source: Life Sci Alliance. 2022 Oct 31;6(1):e202201676. doi: 10.26508/lsa.202201676 (PMC9622424; doi:10.26508/lsa.202201676)
Supplement: Supplementary file 2 [file LSA-2022-01676_TableS2.docx]

Supplementary Table S2: mRNA abundance in *M. maripaludis* JJ constructs

| Plasmid^a^ | Construct | Selenium status^b^ | #Replicates | *bla* copy number per *mcrB* copy number^c^ | ± 95% confidence interval |
| --- | --- | --- | --- | --- | --- |
| pEblaPos2 | Pos 2 | 1 | 4 | 3.87 | 0.52 |
| pEblaPos2 | Pos 2 | 0 | 3 | 2.94 | 2.60 |
| pEblaPos2-S | Pos 2 -S | 1 | 4 | 6.68 | 1.02 |
| pEblaPos2-S | Pos 2 -S | 0 | 4 | 5.52 | 0.68 |
| pEblaPos3 | Pos 3 | 1 | 5 | 6.95 | 2.73 |
| pEblaPos3 | Pos 3 | 0 | 5 | 8.20 | 1.06 |
| pEblaPos3-S | Pos 3 -S | 1 | 4 | 8.44 | 2.13 |
| pEblaPos3-S | Pos 3 -S | 0 | 5 | 10.70 | 1.90 |

a: see Table 1

b: selenite added to the medium (µM)

c: mean
