# Supplementary material for: In vivo probing of SECIS-dependent selenocysteine translation in Archaea
Source: Life Sci Alliance. 2022 Oct 31;6(1):e202201676. doi: 10.26508/lsa.202201676 (PMC9622424; doi:10.26508/lsa.202201676)
Supplement: Supplementary file 3 [file LSA-2022-01676_TableS3.docx]

Supplementary Table S3: Oligonucleotides used in this study

| Name | Sequence 5’→3’ (restriction sites/ overhangs are underlined) | Use |
| --- | --- | --- |
| o_miniFruASECIS_for | CATGGGTCTTGGAAAGGGAACCTTAATGGACCTTGAAAGACA | Cloning of 3’UTR*_fruA_*mini SECIS element |
| o_miniFruASECIS_rev | CATGTGTCTTTCAAGGTCCATTAAGGTTCCCTTTCCAAGACC | Cloning of 3’UTR*_fruA_*mini SECIS element |
| o_mutSECIS_for | CATGGATTCCTGAAAAGGAAAGTCTTGGAAAGGGTCCTAATGGACCCTTTCCAAGACACAAATTAACTTACGGTAATCATAAAAAAATCAA | Cloning of 3’UTR*_fruA_*mut SECIS element |
| o_mutSECIS_rev | CATGTTGATTTTTTTATGATTACCGTAAGTTAATTTGTGTCTTGGAAAGGGTCCATTAGGACCCTTTCCAAGACTTTCCTTTTCAGGAATC | Cloning of 3’UTR*_fruA_*mut SECIS element |
| o_miniFruASECIS_A_C_for | CATGGGTCTTGGAAAGGGAACCTTAATGGCCCTTGAAAGACA | Cloning of 3’UTR*_fruA_*mini_A_C SECIS element, base exchange in bold |
| o_miniFruASECIS_A_C_rev | CATGTGTCTTTCAAGGGCCATTAAGGTTCCCTTTCCAAGACC | Cloning of 3’UTR*_fruA_*mini_A_C SECIS element, base exchange in bold |
| o_bla_qPCR_fw3 | CAGACAACACAGCAGCAAAC | qPCR of *bla* and sequencing of 3’UTR elements |
| o_bla_qPCR_rev3 | TGTGTCTCTTTCGTCGTTAGGG | qPCR of *bla* |
| o_bla_qPCR_rev2 | TGCTGCGATGATTCCTCTTG | cDNA synthesis of *bla* |
| o1555cDNA-2^a^ | GATTACGCCGTCAGCAATAGC | cDNA synthesis of *mcrB*,  base different in JJ (G) compared to S2 (A) in bold |
| o1555RTFor^a^ | CAGTTAACCACGCAGTTGC | qPCR of *mcrB* |
| o1555RTRev^a^ | GTTATCAGCGTTTAATCCTTGG | qPCR of *mcrB* |
| opWLG40-For | CATTGTTAGACCTGCGACAG | Sequencing of reporter in pWLG40NZ-R |
| oGA_blaWLNZR_For | TCTCTTCTTCTTCAGGGAGCTCGAGTTTGTAAAGTGGTAG | Gibson cloning of reporter into pWLG40NZ-R, overlap for reporter construct in bold |
| oGA_blaWLNZR_rev | GGCGTTTTTTATGACCTACAGATCTAATCAATTTTTAAAAATATATAAAAAAAGG | Gibson cloning of reporter into pWLG40NZ-R, overlap with reporter construct in bold |

a: (Stock et al, 2011)
